# Randomized controlled trial of the efficacy of aerobic exercise in reducing metabolic risk in healthy older people: The Hertfordshire Physical Activity Trial

**DOI:** 10.1186/1472-6823-9-15

**Published:** 2009-06-19

**Authors:** Francis M Finucane, Jessica Horton, Lisa R Purslow, David B Savage, Soren Brage, Hervé Besson, Kenneth Horton, Ema De Lucia Rolfe, Alison Sleigh, Stephen J Sharp, Helen J Martin, Avan Aihie Sayer, Cyrus Cooper, Ulf Ekelund, Simon J Griffin, Nicholas J Wareham

**Affiliations:** 1 MRC Epidemiology Unit, Institute of Metabolic Science, Cambridge, UK; 2 Metabolic Research Laboratories, Institute of Metabolic Science, Cambridge, UK; 3 Wolfson Brain Imaging Centre, University of Cambridge, Cambridge, UK; 4 MRC Epidemiology Resource Centre, Southampton, UK

## Abstract

**Background:**

While there are compelling observational data confirming that individuals who exercise are healthier, the efficacy of aerobic exercise interventions to reduce metabolic risk and improve insulin sensitivity in older people has not been fully elucidated. Furthermore, while low birth weight has been shown to predict adverse health outcomes later in life, its influence on the response to aerobic exercise is unknown. Our primary objective is to assess the efficacy of a fully supervised twelve week aerobic exercise intervention in reducing clustered metabolic risk in healthy older adults. A secondary objective is to determine the influence of low birth weight on the response to exercise in this group.

**Methods/Design:**

We aim to recruit 100 participants born between 1931–1939, from the Hertfordshire Cohort Study and randomly assign them to no intervention or to 36 fully supervised one hour sessions on a cycle ergometer, over twelve weeks. Each participant will undergo detailed anthropometric and metabolic assessment pre- and post-intervention, including muscle biopsy, magnetic resonance imaging and spectroscopy, objective measurement of physical activity and sub-maximal fitness testing.

**Discussion:**

Given the extensive phenotypic characterization, this study will provide valuable insights into the mechanisms underlying the beneficial effects of aerobic exercise as well as the efficacy, feasibility and safety of such interventions in this age group.

**Trial Registration:**

Current Controlled Trials: ISRCTN60986572

## Background

Numerous studies have shown that physically active individuals tend to be healthier. Aerobic fitness and habitual physical activity are associated with reduced all-cause mortality, metabolic risk and cardiovascular disease, even in very active individuals [1-4]. Longitudinal cohort studies have confirmed that changes in physical activity are reflected in altered metabolic risk and mortality over time [5]. Trials of interventions to increase physical activity and fitness among older people have tended to be restricted to specific high-risk subgroups. Several trials have shown that lifestyle interventions reduce the risk of progression to diabetes in individuals with impaired glucose tolerance (IGT) and that the beneficial effects of such interventions persist for several years [6-8]. Exercise interventions also reduce cardiovascular and all-cause morbidity and mortality and lead to subjective improvements in quality of life, even in older individuals with established vascular disease [9,10].

While at-risk populations such as those with IGT or vascular disease have been shown to benefit from exercise programmes, the efficacy of such interventions to improve health in the general population remains unproven. In particular, strong evidence that aerobic exercise interventions are beneficial in healthy older people is lacking. Notwithstanding this, public health initiatives encourage people to exercise at least five times per week, irrespective of age or disease risk [11]. While such encouragement seems reasonable given our current knowledge of the benefits of physical activity from observational studies, the evidence base for aerobic exercise interventions in healthy older people ought to be more robust. In practice, old age is often an exclusion criterion for such studies.

Many previous studies that have assessed aerobic exercise interventions have yielded physiologically informative data but have inherent methodological limitations in terms of assessing the efficacy of these interventions per se. Some compare different exercise modalities, without including a true "control" group [12]. Where studies have included controls, often only within-group comparisons are reported, rather than considering changes in outcomes in exercisers in the context of changes in the control group [13]: Using the latter approach allows consideration of any measurement effect and would yield a more valid measure of the efficacy of such interventions. Some efficacy analyses have not included study dropouts, or report only on participants who adhered to the intervention in question, rather than conducting an intention-to-treat analysis [14]. Often, attrition rates from exercise interventions are high, thus reducing power [15]. Indeed, inadequate power is frequently cited as an explanation for negative findings in these studies, even where participant retention has been good [16,17].

Some people respond better than others to aerobic exercise interventions in relation to reductions in body fat, improved fitness and changes in metabolic parameters. Several factors underlie this heterogeneity in the response to exercise. In the diabetes prevention trials, older participants gained more benefit from lifestyle interventions than younger ones did [8,18]. Certain genotypes are known to affect the metabolic response to exercise [19]. Individuals with a family history of diabetes derive more benefit from exercise than those without such a history [20]. Metabolic improvements associated with exercise are more pronounced in those with higher metabolic risk [21]. However the factors that influence the response to aerobic exercise in healthy older individuals have not been fully described.

### Low Birth Weight, Fitness and Metabolic Risk

The thrifty phenotype hypothesis proposes that an adverse intrauterine environment (as manifest by a low birth weight) leads to alterations in the structure and function of various tissues that ultimately confer an increased risk of certain chronic diseases later in life [22]. These alterations arise only when environmental influences occur during certain critical periods of development, when the organism is sensitive to such influences. Studies among individuals exposed to the Dutch "hunger winter" famine of 1944–45 showed that the babies of mothers who were exposed to famine in mid- or late-gestation were born smaller and subsequently had lower glucose tolerance as adults than those whose mothers were only exposed to famine in early gestation or not at all [23]. In animal models, diet restriction in utero or prior to weaning reduces longevity, while dietary restriction after weaning has the opposite effect [24]. Thus, the same environmental exposure can lead to different outcomes, depending on the phase of development in which it occurs. The emergence of programmed changes is modified by adult life factors such as obesity, ageing and physical activity. The highest cardiovascular risk is seen in people who are born small but become overweight as adults [25]. In rural Gambia, even severe malnutrition in early childhood did not lead to adverse metabolic profiles in adults who were lean, fit and consuming a low fat diet [26]. Hence the impact of pre- and post-natal growth on adult disease risk must be considered in the context of adult environmental exposures.

Body fat distribution is a key determinant of cardiovascular risk and foetal growth restriction is known to impact on fat deposition in utero, leading to central adiposity later in life [27,28]. Whether such changes in body fat distribution persist into late adulthood is unknown. Children born small who showed catch-up growth in the first two years of life had a higher percentage body fat and more central adiposity compared to normal birth weight children at age five years [29]. Birth weight predicts subsequent lean mass at different ages, independently of gestational age, and has been shown to correlate with grip strength in older individuals [30,31]. While aerobic fitness is known to reduce cardiovascular risk, it also modulates the association between small size at birth and cardiovascular risk, such that the risk associated with low birth weight is more pronounced in less fit individuals [3]. The association between birth weight and muscle strength persists independently of muscle mass, suggesting that cellular and molecular mechanisms are also modulated by birth weight [32]. Whether aerobic exercise-induced changes in muscle function are differential with respect to birth weight is not known. A previous observational study suggested that exercise has a protective effect against progression to diabetes in low birth weight individuals [33], but higher level evidence from intervention studies is currently lacking.

### Myocellular and hepatic lipid deposition

Skeletal muscle is the major site of insulin mediated glucose disposal. Lipid accumulation in skeletal muscle is strongly implicated in the pathogenesis of insulin resistance and type 2 diabetes [34]. Impaired mitochondrial oxidative phosphorylation may also contribute to this problem [35]. Magnetic resonance spectroscopy studies have suggested that intramyocellular lipid content (IMCL) is a major determinant of muscle insulin sensitivity [36]. Exercise modulates several metabolic pathways in skeletal muscle, enhancing glucose uptake and glycogen synthesis and inducing mitochondrial biogenesis. However, exercise has been shown to increase IMCL, and elevated IMCL levels have been found in endurance trained athletes, despite normal insulin sensitivity [37,38]. Potentially, IMCL is a marker for other lipid intermediates know to suppress insulin sensitivity rather than having a direct insulin desensitising effect in muscle per se [39]. Exercise induced changes in muscle metabolic pathways have previously been shown to markedly improve glucose uptake and mitochondrial function, in all age groups [13]. Whether exercise-induced changes in IMCL in older people are modulated by birth weight has not previously been determined.

Hepatic fat deposition is also a major determinant of insulin sensitivity. While one study in rats suggested that concurrent exercise may prevent liver steatosis induced by a high fat diet, other animal studies suggest there is no exercise effect [40,41]. Similar negative findings have recently emerged in human studies [42]. The effect of aerobic exercise on intrahepatic lipid (IHL) stores in older individuals has not previously been described. MR spectroscopy is considered the best non-invasive method for quantifying intrahepatic lipid content [43]. However, the technique is relatively time consuming and expensive. The search for more practical alternative methods for detecting liver steatosis has begun in earnest, and ultrasound has shown promise in this regard [44]. Whether it is a reliable method for quantifying milder degrees of liver fat deposition remains to be determined. A secondary objective of this study is to assess the reliability, validity and feasibility of ultrasound measures of liver steatosis that may subsequently be applied in larger epidemiological studies.

As individuals get older, body weight declines primarily because of loss of lean tissue [45], while IMCL and liver steatosis tend to increase [46]. The decline in muscle mass results from loss of protein content and individual muscle fibres with a preferential atrophy of type II or fast twitch fibres [47]. The inverse relationship between age and physical activity may account for reduced muscle function in older people [48]. Whether interventions to increase physical activity have an effect on muscle structure and function in this age group has not previously been described. Furthermore, how birth weight affects the relationship between muscle function and physical activity remains unknown.

## Methods/Design

### Study design

This is a single centre, explanatory randomized controlled trial of the efficacy of a fully supervised twelve-week aerobic exercise intervention to reduce overall metabolic risk in healthy older people. Participants are randomized to exercise or control groups following minimisation according to birth weight, percentage body fat, gender and whether or not a muscle biopsy has been performed. The duration of follow-up is 12 weeks from study entry.

### Setting

All baseline and follow-up measures for the trial are conducted at the MRC Epidemiology Unit in the Institute of Metabolic Science in Cambridge. The aerobic exercise intervention is delivered at a gymnasium in Hitchin, Hertfordshire. The study has been approved by the Hertfordshire Research Ethics Committee (LREC reference number 05/Q0201/23).

### Study Population

**Figure 1:**
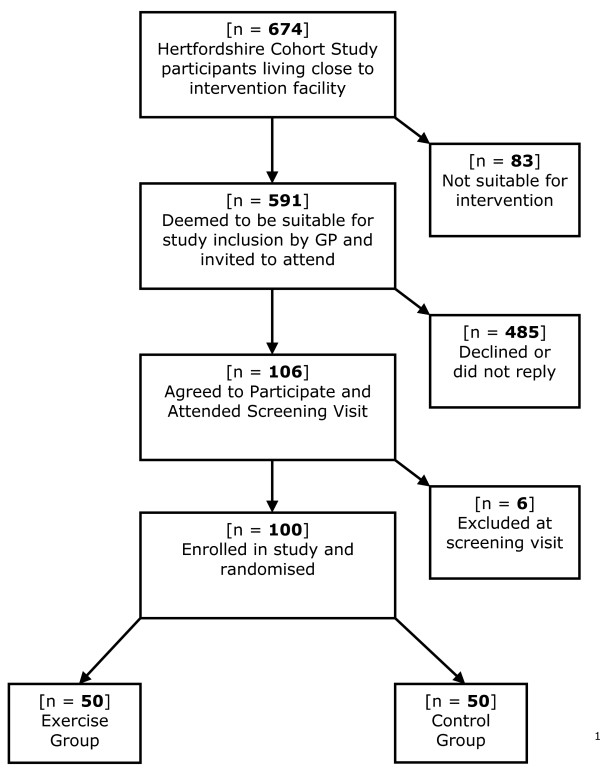
**Flow Chart for the recruitment phase of the Hertfordshire Birth Cohort Physical Activity Trial**.

Participants have been recruited from the Hertfordshire Cohort Study, a unique data resource consisting of men and women born between 1931–39 and still residing there, each of whom had measures of birth weight and growth at one year recorded in infancy [49]. Specifically, we have identified 674 potential study participants within this cohort who live in or near Hitchin, in close proximity to the training facility (see figure 1). We have approached each potential participant's general practitioner to ensure that an invitation to the study is appropriate. Then, an invitation letter and information sheet is despatched, detailing the requirements of study participation and the procedures involved. For those who have agreed to participate, an appointment is made to attend our clinical research facility at Addenbrooke's hospital in Cambridge, after an overnight fast. All procedures are carried out in accordance with the principles of good clinical practice. Fully informed written consent is obtained from each participant prior to testing.

### Exclusion criteria

Individuals with known diabetes, untreated or unstable ischaemic heart disease or any medical condition that would prevent them cycling unaided for at least thirty minutes are excluded from the trial. Those with claustrophobia, cardiac pacemakers or metal implants are precluded from magnetic resonance scanning but have all other study measures taken. Participants taking anticoagulant medication such as warfarin and clopidogrel are excluded from the muscle biopsy procedure.

### Measurements

#### Anthropometry

Weight is measured on a Tanita^® ^scale and height with a Seca^® ^wall-mounted stadiometer. Waist and hip circumferences are measured using a D-loop non-stretch fibreglass tape measure. The waist is defined as the mid-point between the lower costal margin and the level of the superior iliac crests. The hip measurement is taken at the level of the greater trochanter. Blood pressure is measured with an automated oscillometric device (Omron^®^) using the right arm, after participants are seated quietly for five minutes. Three measures are recorded at one-minute intervals. A 12-lead electrocardiogram is performed to exclude occult ischaemic heart disease or cardiac arrhythmia.

#### Dual Energy X-Ray Absorbtiometry (DEXA)

In order to quantify overall and regional changes in body composition, a DEXA scan (Lunar Prodigy Advanced, GE Healthcare) is performed to measure lean mass, fat mass and bone mineral density [50]. Software (enCORE™, GE Healthcare) is used to derive whole body fat percentage, after scan acquisition.

#### Ultrasound

A LOGIQ Book XP^® ^ultrasound device (GE Healthcare) with a 3C-RS curved transducer is used to assess liver parenchymal density relative to renal parenchymal density, as a marker of fatty infiltration. A semi-quantitative grading system is used to define normal echotexture or mild, moderate and severe steatosis. Additionally, ultrasonography is used to quantify abdominal visceral and subcutaneous fat depots. The depth of fat tissue at waist level on the anterior abdominal wall, and the distance between the vertebral bodies and rectus abdominis are taken to represent subcutaneous and visceral fat depots, respectively.

#### Magnetic Resonance Spectroscopy and Imaging

Magnetic resonance measures of IHL, IMCL, visceral- and subcutaneous- adipose tissue (VAT, SCAT) are conducted on a whole body Siemens 3T Tim^® ^Trio scanner at the Wolfson Brain Imaging Centre on the Addenbrooke's campus. For IHL, a ^1^H spectrum is obtained from a voxel of cube length 1.5 cm, located within the posterior aspect of the right lobe of the liver, using the point resolved selective spectroscopy (PRESS) sequence. During this measurement, participants are given breathing instructions with a 7-second cycle, which is designed and gated such that localisation and subsequent data acquisition occur at the end of expiration. Non-water suppressed data are acquired with repetition time (TR) = 7 s, echo time (TE) = 35 ms and averaged over 64 measures. The voxel is positioned to avoid blood vessels and the biliary tree, using T_2_-weighted HASTE transaxial images that are also acquired in the same phase of respiration. The voxel is placed in the same location at the participant's follow-up visit. For IMCL measures, localisation images are acquired in three orthogonal directions through the right lower leg. To aid voxel relocation at the follow-up visit and to ensure the transaxial slices are acquired through the largest bulk of the soleus muscle, the localisation images are planned such that the top of the medial femoral condyle is located in the uppermost slice. The voxel (cube length 1.3 cm) is located within the transaxial slice that contains the most homogenous soleus muscle bulk, avoiding fascial lines and visible fat tissue. A water-suppressed spectrum is obtained using the PRESS sequence with TR = 5 s, TE = 35 ms and 64 averages. All spectra are analysed jMRUI [51] and fitted using the AMARES [52] algorithm with prior knowledge. IHL and IMCL are quantified relative to water and creatine, respectively. Magnetic resonance imaging (MRI) is then used to measure abdominal fat depots in a transverse section. The L4 vertebral body is placed at the isocentre of a 17-slice, T_1_-weighted image with turbo spin echo and water suppression. Transaxial slice thickness is 10 mm with a 2-mm gap. Imaging parameters used are a field of view of 500 × 500 mm, in-plane resolution of 1.3 × 1.3 mm, TR = 400 ms, TE = 21 ms and two averages. Cross-sectional volumes of VAT and SCAT are calculated using a semi-automated method, incorporating a software-generated threshold map (Analyze 7.0, BIR, Mayo Clinic, Rochester MN) which is used in combination with manual input to distinguish the two fat compartments.

#### Blood Samples and Oral Glucose Tolerance Test

A standard 75 g oral glucose tolerance test (OGTT) is performed. Fasting samples are taken for glucose, insulin, C-peptide, liver and lipid profiles. Additional samples are immediately centrifuged and stored at -80°C for subsequent analyses. After ingestion of glucose, further samples are taken every 30 minutes over two hours. The Homeostasis Model Assessment (HOMA) [53] is used to estimate "basal" insulin sensitivity, while the Oral Glucose Insulin Sensitivity (OGIS) model [54] determines "stimulated" sensitivity based on dynamic insulin responses during the OGTT.

#### Muscle Biopsy

Biopsies are taken from the right vastus lateralis muscle with a modified Bergstrom needle (5 mm), using a fully aseptic technique under local anaesthetic. The samples are quickly dried on sterile gauze and then immediately snap frozen in liquid nitrogen for subsequent analysis.

#### Physical Performance Battery and Aerobic Fitness

This validated battery of tests involves assessing five components of basic motor function in older people, namely dominant arm grip strength, time taken to rise from a chair and walk a short distance, repeated chair rises, customary walking speed and balance [55-57]. A sub-maximal exercise test is performed on a cycle ergometer, with a starting workload of 50 Watts (W), increasing by 10 W every minute until 90% of the maximum age-predicted heart rate is achieved or the participant wants to stop. The volume of expired air (VE), carbon dioxide (VCO_2_) and oxygen consumption (VO_2_) is measured at rest for ten minutes prior to exercise, during the test and then for the first five minutes of the recovery phase. An open-circuit respiratory gas analysis system (Jaeger Oxycon Pro^®^), which has been validated elsewhere is used for these measures [58].

#### Objective Measures of Habitual Physical Activity

In order to objectively estimate average daily physical activity energy expenditure (PAEE), each participant wears a combined heart rate and movement sensor (Actiheart^®^, CamNtech, Cambridge UK) continuously for one week [59]. The heart rate response to the sub-maximal exercise test is used for individual calibration of the device [60] and branched equation modelling is then utilised to estimate PAEE [61]. This approach has high validity for estimating the intensity of physical activity [62,63]. Participants wear the Actiheart^® ^for one week after their initial study visit (prior to starting the exercise intervention), then again six weeks later, and finally for a third time after the completion of the last study visit.

#### Questionnaires

A general questionnaire is used to record details of current medications, smoking and alcohol use and dietary patterns. It incorporates the SF-8™ (^©^1998, 1999 QualityMetric Inc., Lincoln, RI), a validated measure of self perceived health status [64]. A detailed family history questionnaire allows the proportion of first degree relatives with diabetes mellitus to be accurately determined. A Recent Physical Activity Questionnaire (RPAQ) is also administered. This quantifies physical activity in four different domains of life (occupation, transportation, home/household and recreational activity) over the preceding month and is modelled on the EPAQ2 questionnaire that our group has validated previously [65]. RPAQ has not previously been validated in older people and so a secondary objective in this study is to do so against the objective measures of physical activity derived from combined heart rate and movement sensing.

### Exercise Intervention

Participants attend the exercise facility for three 1-hour sessions per week over twelve weeks, on Monday, Wednesday and Friday mornings or afternoons. All sessions are fully supervised by the exercise facilitator, and incorporate a warm-up and cool-down period. Participants use upright or recumbent cycle ergometers to achieve an exercise intensity of 50, 60 and 70% of VO_2_max during weeks 1–4, 5–8 and 9–12 of the intervention, respectively. To ensure that the appropriate intensity is maintained during the sessions, heart rate is monitored (Polar Accurex Plus™) and recorded at five minute intervals. For participants on betablockers, the Borg scale [66] is used to assess the intensity of exercise. For those randomized to the exercise intervention, their follow-up visit to the testing facility is scheduled for exactly two days after completion of their final exercise session.

### Statistical methods

#### Outcomes

The primary outcome is the composite metabolic risk score (zMS) measured at follow-up. This risk score is derived by standardising and then summing the following variables to create a standardised score: (Systolic blood pressure + diastolic blood pressure)/2, log 2-hour plasma glucose, log fasting insulin, inverted fasting HDL cholesterol, log triglycerides and waist circumference. Each component variable is standardised using sex-specific means and standard deviations from the larger Hertfordshire Cohort Study population from which these participants were recruited, excluding those with prevalent diagnosed diabetes.

There are several secondary outcome variables, including aerobic fitness, PAEE, total energy expenditure, time spent at different activity intensity levels, blood pressure and resting heart rate. Metabolic secondary outcomes include glucose, insulin and C-Peptide measures during the OGTT, fasting proinsulin: split proinsulin ratios, leptin and adiponectin levels, liver steatosis, IMCL, lipid profiles, indices of insulin sensitivity and glucose tolerance status. Anthropometric outcomes include body weight, fat mass, fat free mass, BMI, waist: hip ratio and visceral: subcutaneous abdominal fat ratios. Additional outcomes include perceived health, perceived quality of life and dietary patterns.

#### Sample Size

With 50 individuals randomized to each of the intervention and control groups, this study will have 85% power to detect a difference in mean zMS risk score of 0.6 standard deviation units between groups at follow-up. This is consistent with our previous observational findings from similar cohorts [2] and assumes that the risk score at follow-up in the control group has a mean of 0 and a standard deviation of 1.

#### Definition of Analysis Populations

For the primary trial efficacy analysis, the intention to treat population includes all participants who were randomized. Then, a per-protocol analysis includes all participants from the control group, but only those intervention participants who completed 85% or more of their prescribed exercise sessions. For certain outcomes, sensitivity analyses will be performed with other subsets of participants excluded, e.g. heart rate responses in those on beta-blockers.

#### Analysis of the primary outcome

The primary analysis compares the composite metabolic risk score at follow-up, adjusted for baseline, between the intervention and control groups. A linear regression model will be used, with follow-up risk score as the outcome and baseline risk score as a covariate.

#### Analysis of the secondary outcomes

The method used to analyse the primary outcome will also be used to analyse the secondary outcomes. For outcomes which do not have a normal distribution, a log transformation will be applied.

#### Missing data

Individuals with a missing value for an outcome variable at follow-up will be excluded from the analysis of that variable. Where the baseline value of a particular outcome is missing, the missing indicator method [67] will be used in sensitivity analyses.

#### Exploratory Analyses

In exploratory analyses, the linear regression model described above will be extended to include an interaction between birth weight and randomized group, to assess whether the effect of the intervention differs according to birth weight. Although this is a randomized trial, there may be some covariates which are not sufficiently well balanced between the exercise and control groups, and where this occurs, these will be considered as potential confounders in subsequent analyses.

## Discussion

Compelling observational data suggest that older people who engage in exercise are fitter and healthier and have lower cardiovascular risk, but higher level evidence that exercise interventions are beneficial in this group is currently lacking. This study will assess the efficacy of such an intervention to improve composite metabolic risk and other endpoints such as glucose tolerance status and insulin sensitivity. The feasibility and safety of a fully supervised, gym-based aerobic exercise programme will also be determined. The extent to which supervised exercise influences habitual physical activity levels will also be explored, as previous work has suggested that compensatory reductions in non-exercise physical activity in older people might negate any benefits [68]. We have not included a dietary modification component to this lifestyle intervention, as to do so may introduce uncertainty as to the mechanisms underlying any observed metabolic improvements. Furthermore, there is some evidence that exercise alone is superior to combined exercise and dietary interventions [69], although other studies suggest combined interventions are best [70]. Observational findings that low birth weight increases subsequent metabolic risk only in adults who are less fit suggests that exercise may have greater benefits in low birth weight individuals [3]. This study is well placed to elucidate any interactions between birth weight and the metabolic response to aerobic exercise, given the relatively large number of participants and the extensive physiological characterization that is involved. This will also allow assessment of other potential determinants of exercise responses, such as diabetes family history. The study will provide valuable opportunities to validate new techniques for measuring body composition and physical activity energy expenditure, both of which are critically important epidemiological outcomes. In addition to a trial efficacy analysis, subsequent cohort analyses will allow the exploration of the effects of birth weight on responses to changes in fitness and body composition. The findings will consolidate our understanding of the putative benefits of exercise in older people, and the mechanisms through which they occur.

## Abbreviations

BMI: Body Mass Index; DEXA: Dual Energy X-ray Absorbtiometry; HCS: Hertfordshire Cohort Study; HDL: High Density Lipoprotein; HOMA: Homeostasis Model Assessment; IGT: Impaired Glucose Tolerance; IHL: Intrahepatic Lipid; IMCL: Intramyocellular Lipid; LDL: Low Density Lipoprotein; MCAR: Missing Completely at Random; MRC: Medical Research Council; MRI: Magnetic Resonance Imaging; OGIS: Oral Glucose Insulin Sensitivity; OGTT: Oral Glucose Tolerance Test; PAEE: Physical Activity Energy Expenditure; PPB: Physical Performance Battery; PRESS: Point Resolved Selective Spectroscopy; RPAQ: Recent Physical Activity Questionnaire; TE: Echo Time (MRI); TR: Repetition Time (MRI); VCO_2_max: Maximal volume of expired carbon dioxide; VE: Volume of Expired air; VO_2_max: Maximal volume of extracted oxygen; zMS: Composite metabolic risk score.

## Competing interests

The authors declare that they have no competing interests.

## Authors' contributions

NJW, SJG, UE: Principal investigators, FMF, DS: Study physicians, JH: Study coordinator, LRP: Protocol development, KH: Exercise facilitator, SB: Physical activity measures, HB: Questionnaire measures, EDR: Anthropometric measures, AS: MR studies, SS: Study statistician, HM: PPB measures, AAS, CC: HCS principal investigators. All authors read and approved the final manuscript. NJW is the paper guarantor.

## Pre-publication history

The pre-publication history for this paper can be accessed here:


